# Bugs Split to Attack and Gamble to Survive

**DOI:** 10.1371/journal.pbio.1001929

**Published:** 2014-08-19

**Authors:** Roland G. Roberts

**Affiliations:** Public Library of Science, Cambridge, United Kingdom

Once there were ten identical monkeys. Their father made them draw lots and gave the winner a long bamboo pole. Each day the monkey with the pole whacks coconuts out of a tree, his brothers catch them and shell them, and they all feed off the proceeds. The monkey carrying the pole finds it quite hard and is normally very tired by the end of the day; over the years, he wonders whether it's worth the hassle. But one day there's an almighty flood. The monkey with the pole is lagging behind as usual and watches his nine brothers drown in the distance. When the flood abates, he walks on, finds a mate, and eventually passes the pole on to one of his ten identical children.

Bacterial populations grow rapidly and asexually, generating millions of genetically identical progeny. Traditionally, it has been assumed that if these bugs are placed in a uniform environment, then they'll behave identically—nature and nurture are invariant, so there is neither motivation nor mechanism for diversity. Increasingly, however, we're appreciating that there are many intrinsic advantages to diversity, and nature can readily exploit this by using stochastic mechanisms to program populations of organisms to vary despite genetic and environmental uniformity. As with the monkeys, however, there are several ways in which it can pay to be different.

One obvious way in which diversity of phenotype can yield dividends is by allowing specialization: “division of labor.” The unpleasant human pathogen *Salmonella* Typhimurium is one bug that's already known to do this; a small proportion of a genetically identical horde of invading bacteria express high levels of virulence factors called *ttss-1*, while the majority produce only low levels. The *ttss-1* genes encode Type III Secretory System 1, a large and complex molecular “hypodermic needle” that allows the high-expressing “T+” bugs to inject the host cells full of effector proteins. These proteins in turn manipulate the host cells in a range of ways that favor proliferation by the T+ bacteria's more numerous low *ttss-1*-expressing “T−” brethren, thereby promoting virulence.

**Figure 1:**
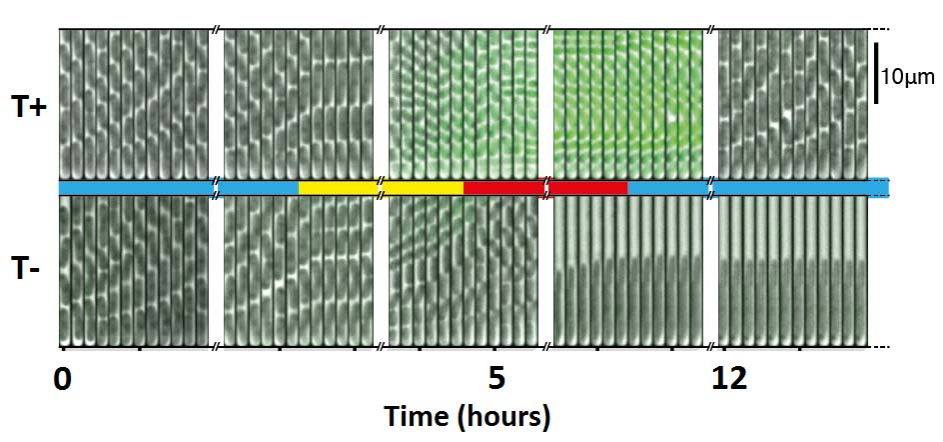
Bugs in tubes. A 13-hour time-lapse montage of images from the microfluidics device used for this study. The bottom of each tube is closed so that as bugs divide they move up the tube and are shed at the top. The blue bar in the middle indicates normal medium, the yellow bar indicates the use of depleted medium to induce the choice between T+ and T− phenotype, and the red bar indicates the challenge with the antibiotic ciprofloxacin. In the top row of images, the cell at the bottom of the tube has a T+ phenotype, as indicated by the green fluorescent protein reporter, and survives the antibiotic. In the bottom row, the corresponding cell is T− and perishes.

A paper just published in *PLOS Biology* by Markus Arnoldini, Martin Ackermann, and colleagues adds a biologically intriguing and potentially clinically important twist to this touching tale of cooperation in *Salmonella*. They start from the observation that the T+ subpopulation seems to grow more slowly, and the authors check this in detail using an elegant high-throughput single-cell method based on time-lapse movies of a microfluidic system and bacteria containing a fluorescent reporter of *ttss-1* expression (Figure 1). Slow growth is often associated with tolerance of antibiotics, however, so the authors ask the direct question of whether the T+ cells are better able to survive the ravages of a dose of ciprofloxacin. They were, and the authors then confirm that survival depends on the ability to turn on *ttss-1* and does not involve the emergence of drug resistance by mutation. The T+ bugs were also more tolerant to kanamycin, a structurally and functionally unrelated antibiotic, and even survived high, clinically relevant doses of both drugs.

The assumption was that the costly expression of abundant protein (in this case, *ttss-1*) caused slow growth, and slow growth caused tolerance. The authors tested this mechanistic chain by artificially slowing the growth of bugs that couldn't express ttss-1 and by gratuitously expressing high levels of an arbitrary protein, LacZ. Both experiments made bugs more tolerant to antibiotics.

Like the monkey's pole, *ttss-1* expression is therefore the agent both of a continuing cooperative behavior (virulence) and a burdensome bet hedge that pays off (tolerance) when disaster strikes. A potentially alarming consequence of this is that treating an infection with antibiotics might inadvertently select for virulence by preferentially culling nonvirulent (T−) cells. To examine whether this is possible, the authors set up a competition between normal *Salmonella* (which can switch stochastically between T+ and T− states) and a genetically avirulent one which is always T−. In the absence of antibiotic, the unburdened avirulent bugs win, but if antibiotic is present, the hedged bet pays off, and the switchers take over.

The study therefore shows that two fundamental drivers of cellular heterogeneity—division-of-labor and bet hedging—can be intricately intertwined by the sharing of a common agent. The authors wonder whether this entanglement means that bet hedging might serve to prevent cheater mutants (those which can't switch to a T+ phenotype but freeload off those that can) from subverting the division-of-labor system. Crucially, when the heterogeneous traits involved are drug tolerance and virulence, then unfortunate clinical consequences may arise from the peculiar confluence of two facts—that expression of virulence proteins is expensive enough to slow cellular growth and that slow cellular growth helps bacteria to weather the presence of antibiotics. Clearly we need a better understanding of this unforeseen potential downside of antibiotic treatment; we don't want to select for killers when we aim to cure.

**Arnoldini M, Vizcarra IA, Peña-Miller R, Stocker N, Diard M, et al. (2014) Bistable Expression of Virulence Genes in** ***Salmonella*** **Leads to the Formation of an Antibiotic-Tolerant Subpopulation.** doi:10.1371/journal.pbio.1001928

